# Marine Biotechnology

**DOI:** 10.1155/2011/639140

**Published:** 2011-11-02

**Authors:** Song Qin, W. E. G. Müller, Edwin L. Cooper

**Affiliations:** ^1^Chinese Academy of Sciences, Beijing 100864, China; ^2^Johannes Gutenberg University of Mainz, 55122 Mainz, Germany; ^3^University of California, Los Angeles, Los Angeles, CA 90095, USA

Modern marine biotechnology has been developing rapidly since the 1980s. There are promising and exciting achievements in biochemistry, genetics, genomics, aquaculture, bioenergy, and other related fields, beginning with genetic recombinant technology as applied to marine algae. Marine biotechnology clearly incorporates enormous social and economic benefits, thus providing a foundation for problems related to food as exemplified by ocean farming. Marine biotechnology is relatively young but reveals enormously vigorous and powerful applications. These include approaches of marine biotechnology from genomics to marine aquaculture and from genomic engineering to ocean farming.

For more specific health benefits to humans, applicable pharmaceuticals are likely to emerge as more natural products are shown to be effective. This special issue is devoted to marine biotechnology. This issue contains 33 papers that pertain to our original goals and include twelve topics: *algal biotechnology; marine microbiology; marine drugs; genomics, proteomics, and metabolomics in marine biotechnology; marine bioactive compounds; marine bioproducts; biomaterials and nanobiotechnology; biomineralization, biomineral, and biomarker; oceans and human health; drug discovery; biotechnology and development; pharmacologic mechanisms*.

For the convenience of the readers papers have generally been arranged according to the genus and species of the organism from which products have been derived. Briefly here are the descriptions: papers 1–6: bacteria; 7–13: algae; 14-15: fungi; 16–19: microbial libraries; 20–25: complex higher invertebrates; 26–27: sponges; 28: crabs; 29–31: fish; 32: humans. To round out the entire list, the final paper 33 focuses on metagenomic libraries.

The inspiration for this special issue has grown from early contributions to Evidence-Based Complementary and Alternative Medicine. The term bioprospecting was introduced by Müeller and later expanded by Cooper. The prefix bio signifies life while prospecting is defined as “an expectation, a possibility, a chance of success or advancement” to explore in search of something. When put together, they fit the kind of searches that have been explored in this issue. Compounds such as bioactive proteins (pore-forming protein and tachylectin) from sponges may be used for antibacterial activity while skeletal elements such as biosilica serve as blueprints for new biomaterials applicable to biomedicine. And since that time, other papers have emphasized the importance of the biosphere (both terrestrial and aquatic) as a vital store for expanding the repertoire of potential products that can ultimately be of use as sources of food and pharmaceuticals.

In addition to a search for marine natural products, clearly the symposium and now the resulting issue have underscored the need for international cooperation as we continue to search for products with valuable applications to human health. The aim of this issue was to present recent advances in the discovery and development of marine natural products, which has laid the foundation for the synthesis of proteins, drugs, and other bioproducts with special functions. Manuscripts in this special issue covered several aspects of recent developments in the vast field of marine biotechnology. Other manuscripts highlight previous investigations but are orientated towards the more practical concerns of application rather than simple analysis of exotic marine animals themselves. The firm grounding of biology and the resulting amalgam of molecules derived from invertebrate immune systems lies at the forefront of new scientific discovery and societal advancement. 

